# Transactions of Chicago Society of Physicians and Surgeons

**Published:** 1873-12

**Authors:** Plym. S. Hayes

**Affiliations:** Chicago


Article IV—*Transactions of the Chicago Society of Physicians and Surgeons. Meeting of November* 10, 1873. Reported by Plym. S. Hayes, M.D., Chicago.

This Society was organized after the fire for the benefit of physicians resident in the southern portion of the city; but has since added to its number those practicing in both the North and West Divisions, and has increased to such an extent that it was considered desirable to hold its meetings in a more central location. For this reason the meeting was called in the Grand Pacific Hotel.

The Society was called to order at 8 o'clock P. M., by the Vice-President, in the absence of the President, Dr. A. Fisher. There were forty present, including the President, Secretary, and several members of the Chicago Medical Society, and some representatives of the Illinois State Microscopical Society.

The Chairman of the committee on a permanent place of meeting, reported on the subject in full. This report was accepted the committee discharged, and further consideration of the subject deferred to the next regular meeting.

Dr. John Bartlett then read a paper entitled, "*On a Marsh Plant from the Mississippi River Ague Bottoms; with a consideration of its genetic relation to Malarial Diseases.*"

Dr. Bartlett then described a marsh plant, the growth of which he discovered to be simultaneous with the appearance of malarial fevers, containing cells of exceeding minuteness, which possessed the power of rapid multiplication. These had been detected by Dr. Bartlett, in his own blood, and in that of several ague patients; and had been seen by him multiplying with great rapidity, and producing products similar to those recognized as peculiar features of the plant, in the blood, the urine and the saliva. On the conclusion of the reading, the members of the Society and invited guests inspected the microscopic preparations, colored drawings of the plant, and specimens of the plants themselves, growing in their native soil, which were intended to illustrate the reading.

The Society then adjourned, to meet again in the Grand Pacific Hotel, November 24, 1873.

